# Community structure of fungal pathogens causing spikelet rot disease of naked oat from different ecological regions of China

**DOI:** 10.1038/s41598-020-80273-6

**Published:** 2021-01-13

**Authors:** Longlong Liu, Mingchuan Ma, Zhang Liu, Lijun Zhang, Jianping Zhou

**Affiliations:** Key Laboratory of Crop Gene Resources and Germplasm Enhancement on Loess Plateau, Center for Agricultural Genetic Resources Research, Shanxi Agricultural University, Taiyuan, 030031 China

**Keywords:** Microbial communities, Sequencing

## Abstract

Spikelet rot disease (SRD) is an emerging disease of the grain surface of naked oat in China that affects both grain yield and quality. The typical symptom is discoloration from the black structures of the causal fungi. Here, we investigated the fungal communities on the grain surfaces of cultivar Bayou 13 grown in ten ecological oat-producing regions of China, to identify the main pathogens of naked oat SRD. Our results showed that the growth of *Alternaria* spp. and *Davidiella* spp. exhibited a competitive relationship and was mainly affected by the elevations of all 10 ecological regions. The dominant pathogens were *Davidiella* spp. in Shannan Prefecture in Tibet and Haidong Prefecture in Qinghai Province and *Alternaria* spp. in the other eight regions. The ratios of black pathogens of interest to all pathogens in Shannan Prefecture and Haidong Prefecture were significantly lower than those of the other eight regions, thus indicating that SRD mainly occurred in regions below 2000 m (elevation). We isolated black fungal pathogens from grain surfaces and deduced that they were *Alternaria* spp. by sequence comparison. The blackened appearance of the grain surfaces was more evident under spray inoculation with a spore suspension of *Alternaria* than under the control in greenhouse experiments. The recovered pathogen was the same as the pathogen used for inoculation. We thus concluded that *Alternaria* alone causes naked oat SRD and mainly infects naked oat in regions below 2000 m, which provides a basis for the recognition and management of SRD of naked oat.

## Introduction

Oat (*Avena sativa*) is one of the most important cereal crops in the world and is cultured in all agricultural regions of the world with a moderate climate^1^. The hexaploid cultivated species *A*. *sativa* is represented by both hulled (*A*. *sativa* subsp. *sativa* L.) and naked forms (*A. sativa* subsp. *nudisativa* (Husnot.) Rod. et Sold.)^2^, which are believed to have originated in China^3^. The yield and composition of nutritional and functional active ingredients were higher in naked oat than in hulled oat^4,5^. Naked oat is the main cultivated oat species in China, accounting for 92% of the total area planted in oat (106.7 hm^2^ of 120.0 hm^2^)^6^. Naked oat is mainly distributed in arid and cold regions, including Inner Mongolia, Yunnan Province, Gansu Province, Sichuan Province, Hebei Province, Shanxi Province, Qinghai Province, Jilin Province, and Tibet^7^. In these regions, rainfall is scarce and mostly concentrated from July to September. The occurrence of continuous rainfall during the harvest season leads to high moisture and warm temperatures being important factors in the spread of plant diseases^8^. In this period, many fungi on glumes and panicles of weakened plants or moribund tissues of cereal crops result in grain discoloration^9^. Extensive occurrence of spikelet rot disease (SRD, Supplementary Figure S1) on oat grains in fields is a result of rainfall just before harvest and is a regularly observed phenomenon^10^.

SRD of naked oat is characterized by black fungal discoloration, especially on the surface of the kernels as well as beneath the pericarp of the grain ears^11^. This discoloration is mainly considered a blemish of the grain but not a factor affecting quality^10^. This disease has generally received comparatively little attention and is considered a minor disease that does not warrant specific control measures^10^ when compared with oat diseases such as rusts, blights, smuts, spots, and culm and root rots^1,12–14^. Recently, the most general usage of naked oat has been for livestock feed, but oat consumption as a human food has recently increased, perhaps due to the reported health benefits arising from the nutritional value of the grains^14^. However, fungal infection of grains in the field may result in yield nutritive value reduction and the production of toxins that are harmful to humans and animals^10,15,16^. Thus, a high grain quality is of great significance to the safe production of naked oat products.

Most studies have focused on rice SRD, and one of its features is that it is not associated with one but four different pathogens^17–21^. Previous reports have shown that many fungi, including members of the genera *Alternaria* and *Fusarium,* are associated with grain contamination of oat, wheat and barley^10,15,22–26^. Species of the genera *Alternaria* and *Fusarium* are plant pathogens and are commonly found as surface contaminants on grains, in soil and on dead or dying plant tissues^10^. With sufficient surface moisture, the fungal spores present on the kernels can germinate and grow. With sufficient fungal growth, the husk can become discolored. However, *Fusarium* spp. were not observed on normal and discolored grains of naked oat in China, while more *Alternaria* spp. were isolated from discolored grains than from normal grains^27^. Currently, there are no systematic reports on SRD on naked oat, but it is considered an increasingly important disease of naked oat in China. Such a lack of knowledge hampers the development of suitable disease risk forecasts and control measures.

The species abundance and composition of the microbiota are important factors in determining the quality of the grain^15^. Throughout the growing season, weather conditions such as temperature and humidity influence the distribution of the infecting fungal species and lead to geographical variation in the species distribution^28–31^. As the main cultivar of naked oat in China, Bayou 13 is planted in different ecological regions, including Tibet, Shanxi Province, Gansu Province, Inner Mongolia, Qinghai Province and Hebei Province. Therefore, in this study, we investigated fungal pathogens found on the surfaces of grains produced in these regions associated with typical SRD symptoms, identified the associated black pathogens, and carried out greenhouse infection experiments to determine the main pathogen and pathogen distribution for this disease.

## Results

### Metagenomic sequence characteristics of fungal pathogens on grains from different ecological regions

Metagenomic sequencing of the fungal communities on the grain surfaces of Bayou 13 obtained from 10 ecological regions during the harvest season yielded 1,458,785 valid sequences, with 99.29% (1,448,477) being high-quality sequences. The proportion of high-quality sequences was more than 97% for all regions. The samples from Shannan Prefecture produced the fewest valid and high-quality sequences (114,171 and 113,867, respectively). The samples from Dingxi City in Gansu yielded the most valid and high-quality sequences (200,080 and 199,194, respectively) (Table 1). The sequences had lengths ranging from 173 to 283 bp, with 480,675 (33.18%) sequences having a length of 260 bp, 269,758 (18.62%) sequences having a length of 250 bp, 157,147 (10.85%) sequences having a length of 251 bp, 145,130 (10.02%) sequences having a length of 249 bp, and 113,720 (7.85%) sequences having a length of 234 bp. For the vast majority of the sequence lengths, the number of sequences was > 1000.

**Table 1:** Valid and high-quality sequences obtained from metagenomic sequencing of fungal pathogens on the surfaces of Bayou 13 grains from 10 ecological regions.

Ecological region	Valid sequences	High-quality sequences	High-quality sequences/valid sequences (%)
Kelan County, Shanxi Province	158,447	157,375	99.32
Dingxi City, Gansu Province	200,080	199,194	99.56
Datong City, Shanxi Province	125,628	125,155	99.62
Chifeng City, Inner Mongolia	185,197	182,857	98.74
Jining City, Inner Mongolia	133,994	133,281	99.47
Hinggan League, Inner Mongolia	120,721	119,073	98.63
Zhangjiakou City, Hebei Province	146,646	146,049	99.59
Baicheng City, Jilin Province	153,187	151,395	98.83
Shannan Prefecture, Tibet	114,171	113,867	99.73
Haidong Prefecture, Qinghai Province	120,714	120,231	99.60
Total	1,458,785	1,448,477	99.29

### Species diversity and richness of fungal pathogens on grains from different ecological regions

In total, 1880 OTUs were produced from the sequencing of fungal pathogens on grains from 10 regions. When the calculated diversity indices were compared, the Chao1 and ACE indices were similar to the OTUs. The highest Chao1 and ACE index values were observed in samples from Dingxi City, and the lowest Chao1 and ACE index values were observed in samples from Datong City. The highest values for the Shannon indices were observed in samples from Baicheng City, and the lowest values were observed in samples from Shannan Prefecture, Tibet (Table 2).

**Table 2:** Diversity indices of fungal pathogens on grains of Bayou 13 from 10 ecological regions.

Ecological region	OTUs	Chao1 estimator	ACE estimator	Shannon index
Kelan County, Shanxi Province	172	162.821	165.875	2.770
Dingxi City, Gansu Province	188	201.954	201.729	2.121
Datong City, Shanxi Province	156	138.167	150.132	1.928
Chifeng City, Inner Mongolia	210	197.431	201.655	2.777
Jining City, Inner Mongolia	180	176.888	181.924	2.373
Hinggan League, Inner Mongolia	162	161.372	158.034	1.790
Zhangjiakou City, Hebei Province	174	185.212	171.447	2.432
Baicheng City, Jilin Province	182	189.288	174.731	3.131
Shannan Prefecture, Tibet	152	151.611	165.347	1.779
Haidong Prefecture, Qinghai Province	155	146.633	159.059	2.425

**Figure 1:**
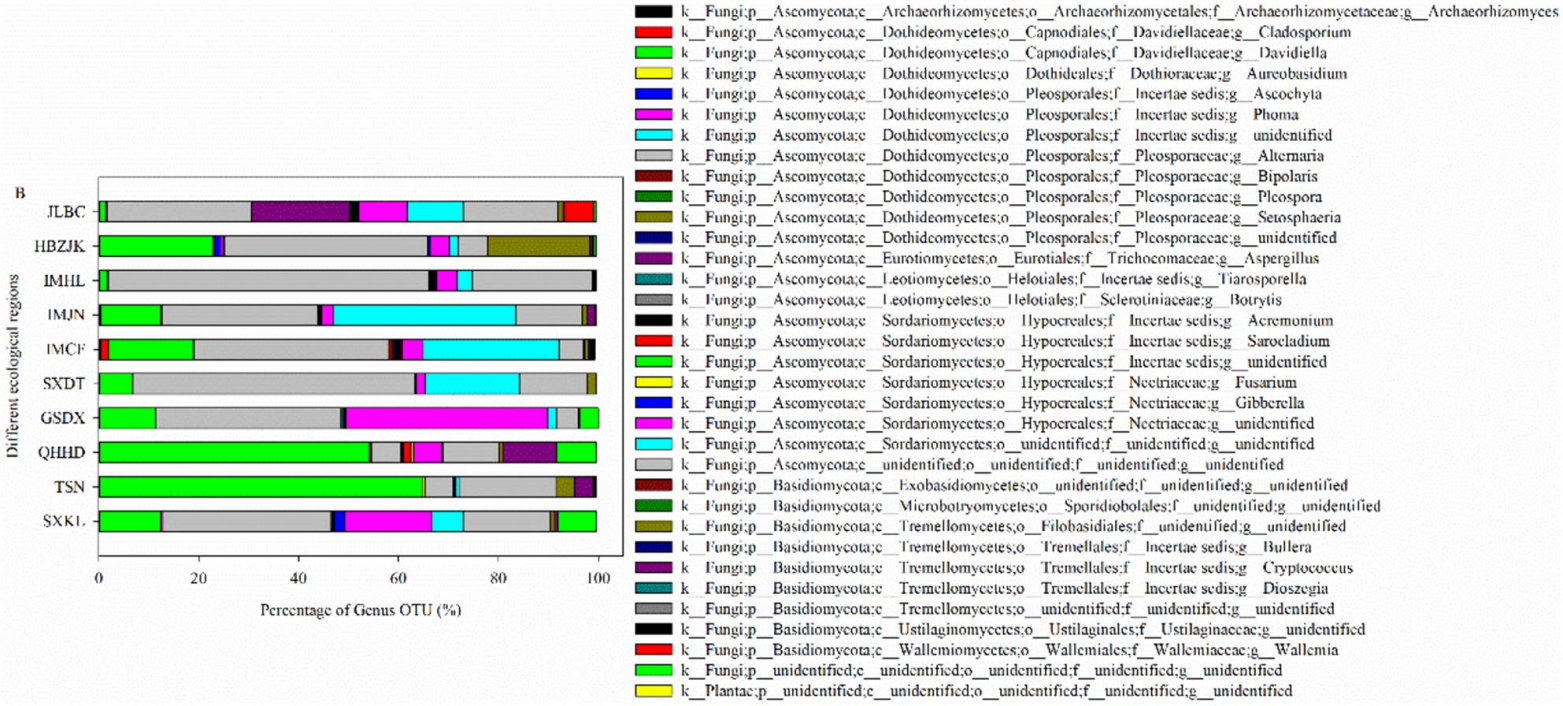
Genus-level taxonomic composition of fungal pathogens on grain surfaces of Bayou 13 from 10 ecological regions. SXKL, TSN, QHHD, SXDT, GSDX, IMCF, IMJN, IMHL, HBZJK, and JLBC indicate Kelan County in Shanxi Province; Shannan Prefecture in Tibet; Haidong Prefecture in Qinghai Province; Datong City in Shanxi Province; Dingxi City in Gansu Province; Chifeng City, Jining City, and Hinggan League in Inner Mongolia; Zhangjiakou City in Hebei Province; and Baicheng City in Jilin Province, respectively.

In total, 69 genera were detected on grain surface samples, with the dominant pathogens belonging to the genera *Alternaria* (5.4–64.0%) and *Davidiella* (1.4–64.6%). *Alternaria* was the dominant species in the Kelan (33.6%), Dingxi (36.7%), Datong (56.3%), Chifeng (37.7%), Jining (31.1%), Hinggan League (64.0%), Zhangjiakou (40.5%), and Baicheng (28.8%) regions. *Davidiella* was the dominant species in Shannan Prefecture (64.4%) and Haidong Prefecture (53.9%). Judging from the proportions, *Alternaria* was much more common than *Davidiella* in all regions (Fig. 1).

**Figure 2:**
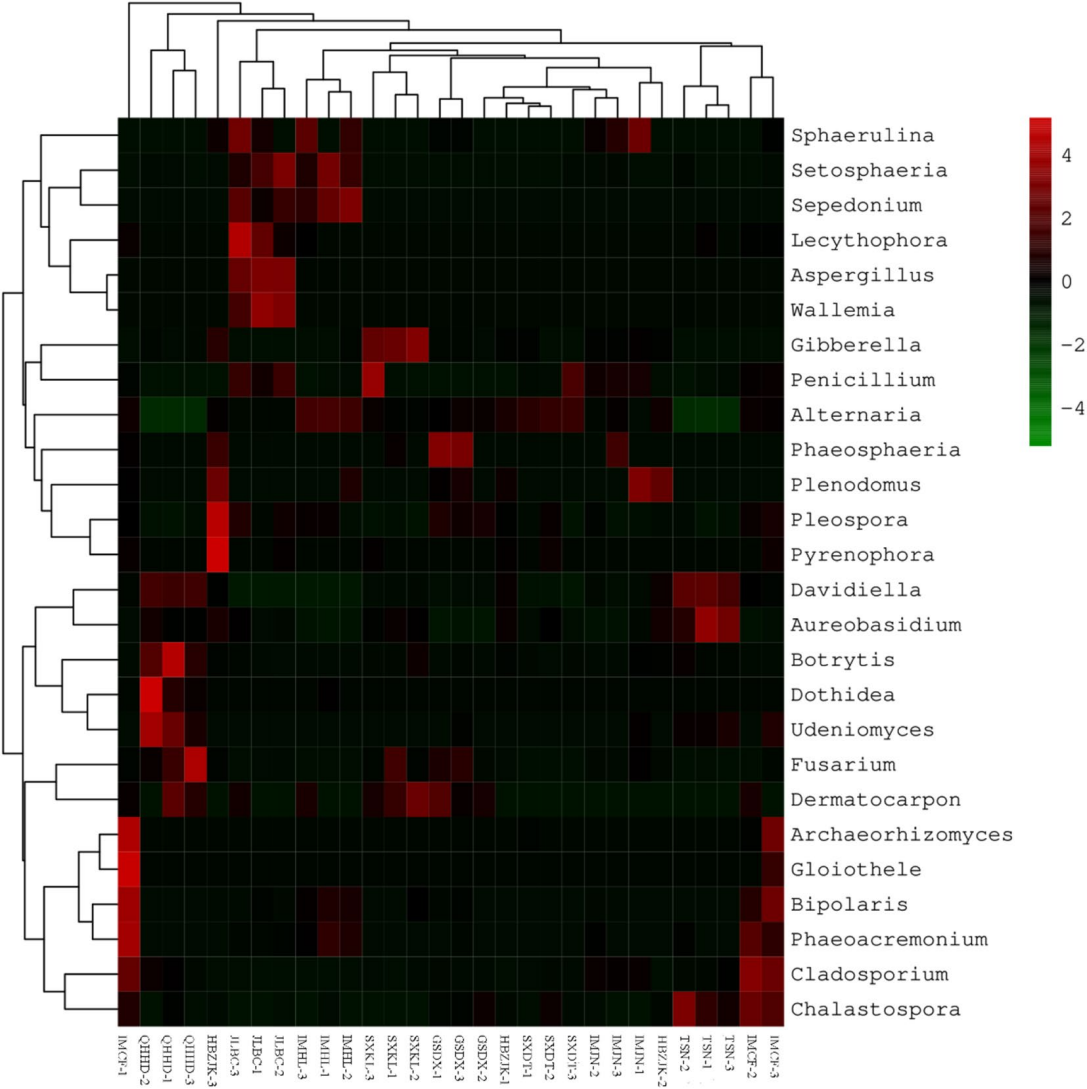
16S rDNA sequencing reveals the relationship and classification of fungal pathogens on the surfaces of Bayou 13 grains from 10 ecological regions at the genus level. The weighted UniFrac UPGMA tree is based on 13 fungal genera. The heat map shows the relative abundance of the 13 genera within each sample that were most abundant in the entire dataset. The abundance data were normalized by range-scaling each class to Log_2_. SXKL, TSN, QHHD, SXDT, GSDX, IMCF, IMJN, IMHL, HBZJK and JLBC indicate Kelan County in Shanxi Province; Shannan Prefecture in Tibet; Haidong Prefecture in Qinghai Province; Datong City in Shanxi Province; Dingxi City in Gansu Province; Chifeng City, Jining City, and Hinggan League in Inner Mongolia; Zhangjiakou City in Hebei Province; and Baicheng City in Jilin Province, respectively.

Hierarchical cluster analysis at the genus level revealed that 13 genera, including *Alternaria*, *Pyrenophora,* and *Gibberella,* belonged to one cluster, and 13 others, including *Davidiella*, *Botrytis*, and *Bipolaris*, belonged to another cluster. When region-based clustering was performed, it was found that Haidong Prefecture formed a cluster of its own, and all other regions formed another cluster (Fig. 2).

**Figure 3:**
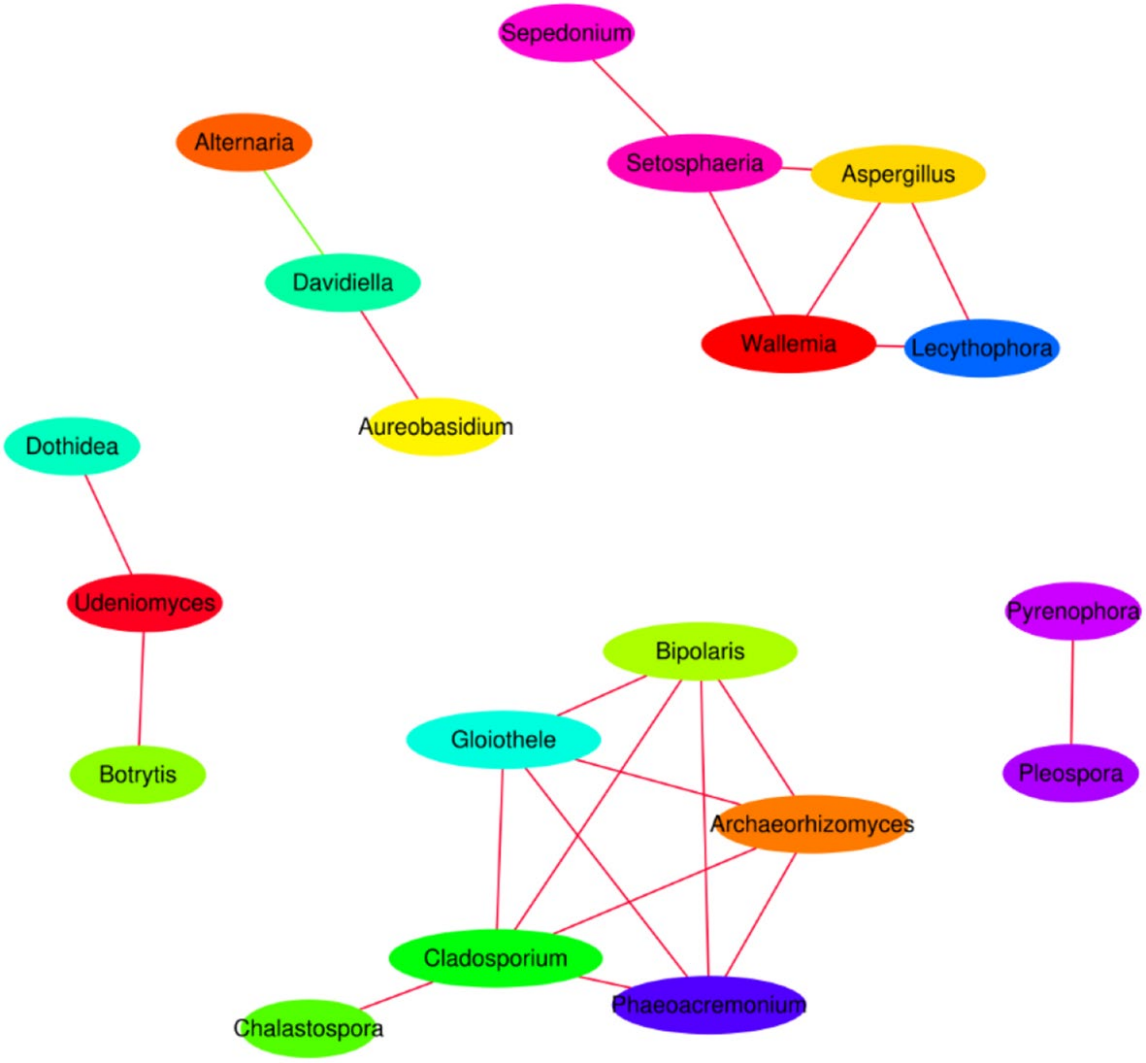
Network analyses at the genus level according to the abundance of fungal pathogens on the surface of Bayou 13 grains from 10 ecological regions. Network analyses of Spearman correlation coefficients reveal the cooccurrence patterns among genera. Different colors represent different genera. Edges (lines) between nodes are colored red for positive correlations between genera and green for negative correlations between genera.

Based on the richness of each species in the various samples, the correlations among species were calculated using the richness information at the genus level for the top 50 species. A correlation network consisting of significantly correlated species was constructed for the prediction of interactions among species. From Fig. 3, it can be seen that, with the exception of one negative correlation (i.e., competitive relationship), all other correlations between *Davidiella* and *Alternaria* were positive (i.e., collaborative relationships).

By eliminating these six indices, we found one significant single linear relationship between the percentage of *Alternaria* spp. (*y*) and elevation (x): *y* = 53.272 − 0.013*x*, *R*^2^ = 0.568, (*F*_1,9_ = 10.54, *P* < 0.05), and there were significant differences in elevation (*t* = − 3.246, *P*_*x*_ < 0.05); another relationship was found between the percentage of *Davidiella* spp. (*y*) and elevation (x): *y* = − 5.59 + 0.018*x*, *R*^2^ = 0.805, (*F*_1,9_ = 33.064, *P* < 0.05), and there were significant differences in elevation (*t* = 5.75, *P*_*x*_ < 0.05).

### Culture of fungal pathogens from grains from different ecological regions

**Figure 4:**
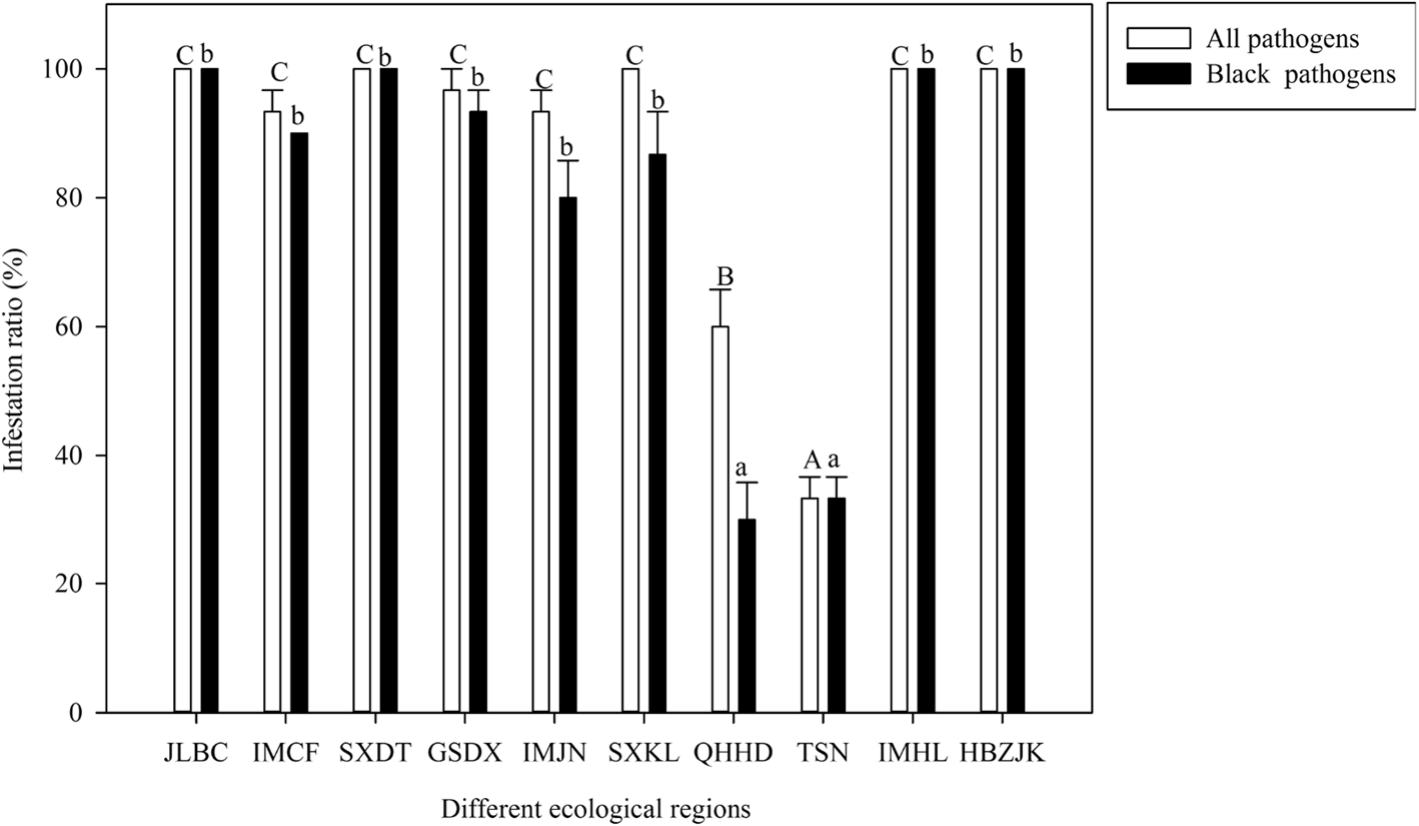
The comparison of infections by fungal pathogens after 5 days of culture on PDA on the surface of Bayou 13 grains from 10 ecological regions, China. The white and black columns represent the means for all pathogens and black pathogens, respectively, and the bars represent the SE. Different uppercase letters and lowercase letters above the columns indicate significant differences within the ten regions (least significant difference test, *P* < 0.05) for all pathogens and black pathogens, respectively. SXKL, TSN, QHHD, SXDT, GSDX, IMCF, IMJN, IMHL, HBZJK and JLBC indicate Kelan County in Shanxi Province; Shannan Prefecture in Tibet; Haidong Prefecture in Qinghai Province; Datong City in Shanxi Province; Dingxi City in Gansu Province; Chifeng City, Jining City, and Hinggan League in Inner Mongolia; Zhangjiakou City in Hebei Province; and Baicheng City in Jilin Province, respectively.

Based on the information obtained on the fungal pathogen community (Supplementary Table S2, Supplementary Figure S2), the infestation ratio of all pathogens on the grains from Haidong Prefecture in Qinghai Province was significantly higher than that from Shannan Prefecture in Tibet and significantly lower than that in the other regions (*F* = 64.96, df = 29.9, *P* < 0.05) (Fig. 4). Moreover, the infestation ratios of black pathogens from Shannan Prefecture in Tibet and Shannan and Haidong Prefectures were significantly lower than those from the other regions (*F* = 49.557, df = 29.9, *P* < 0.05) (Fig. 4).

### Verification of SRD-causing fungal pathogens on grain surfaces

**Figure 5:**
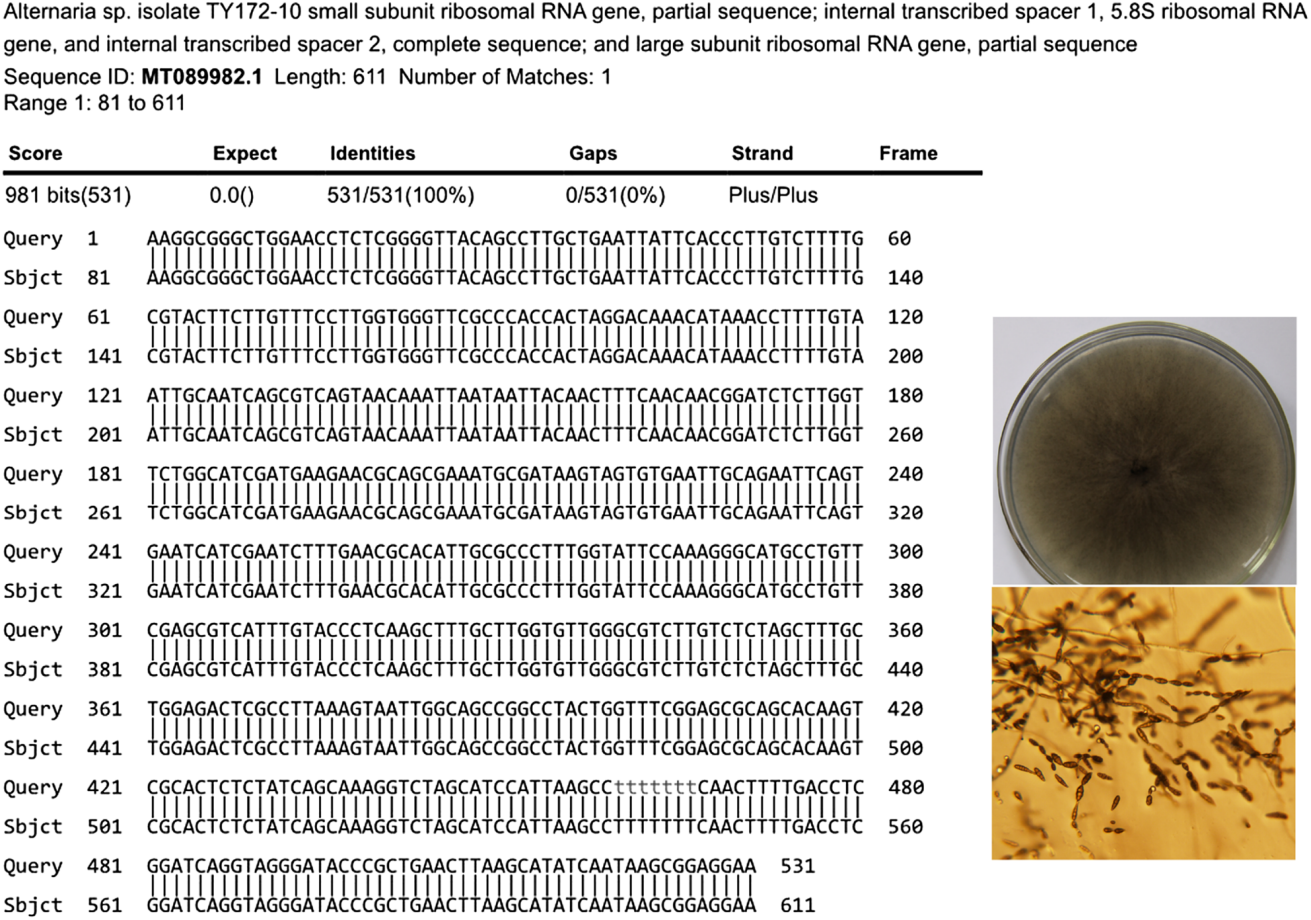
Molecular identification and BLAST comparison of *Alternaria* spp. on black spots of grains from three plates for each of the 10 ecological regions. A plate containing 10 mildewed grains of Bayou 13 cultured on PDA medium for 5 days.

**Figure 6:**
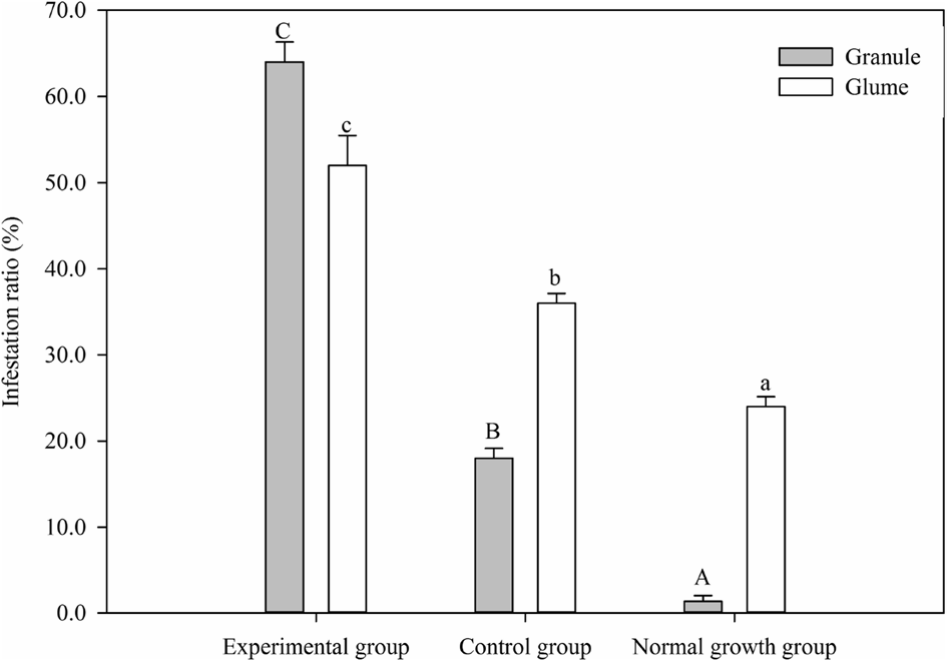
The comparison of infections by *Alternaria* spp. after 5 days of culture on PDA in the experimental group (2 weeks spraying with spore suspension of *Alternaria*), control group (2 weeks spraying with water containing 0.1% Tween-20), and normal growth group (untreated) for either glumes or grains of Bayou 13 from Kelan County in Shanxi Province, China. The gray and white columns represent the means for granules and glumes, respectively, and the bars represent the SE. Different uppercase and lowercase letters above the columns indicate significant differences within the three groups (least significant difference test, *P* < 0.05) for granules and glumes, respectively.

The black pathogen strains purified on the grain surfaces from the 10 ecological regions were identified as *Alternaria* spp. through sequencing analysis and morphological identification (Fig. 5). After the granules and glumes of the three groups had been cultured on PDA medium for 5 days, greenhouse experiments were performed, and the infestation ratios of granules (*F* = 142.855, df = 8.6, *P* < 0.05) and glumes (*F* = 49.258, df = 8.6, *P* < 0.05) in the control group were significantly higher than those in the normal growth group (untreated) and lower than those in the experimental group (Fig. 6). The black pathogen strains were again isolated from the granules and glumes of the experimental group. The isolates were observed to be consistent with the original isolates, thus fulfilling Koch's postulates.

## Discussion

Through the investigation of the community structure of fungal pathogens on the surface of Bayou 13 oat grains from 10 ecological regions, our results showed that the fungal pathogens mainly belonged to 69 genera of Ascomycota and Basidiomycota, with the dominant pathogens belonging to *Alternaria* spp. and *Davidiella* spp. The same results were found in *Lolium perenne* and *Trifolium repens*^32^. These two fungal genera are widely distributed in different proportions in plants, aquatic ecosystems and soils^33–35^. Ascomycetes are more easily amplified than Basidiomycota with the ITS2 subsite^36^, while *Davidiella* was the dominant genus amplified with the ITS2 region^37^. Furthermore, we observed that these two genera exhibited a competitive relationship. A negative interaction between *Fusarium* spp. and *Alternaria* spp. was found on the surface of wheat and barley grains of reduced quality^10,22^. Fungal distribution is mainly affected by fungal interactions such as spatial competition^38^. This may explain why, except for Shannan Prefecture and Haidong Prefecture, the proportion of Ascomycota in the other eight regions was significantly higher than that of Basidiomycota, and *Alternaria* spp. were the dominant fungi in the eight regions. In addition to being proven with plate culture, fungal cooccurrence has been investigated by metagenomic approaches and network analysis^39,40^. We also found that *Alternaria* and *Davidiella* spp. were negatively and positively correlated with elevation, respectively. Elevational gradients exert a strong influence on the relationships among crops and their microbiota in alpine regions^41,42^. This result thus indicated that elevation mainly affected the relative occurrence of *Alternaria* and *Davidiella* spp., presenting an inverse relationship with blackened surfaces of Chinese naked oat grains.

On the other hand, the infestation ratios on grains from Shannan Prefecture and Haidong Prefecture were significantly lower than those from the other eight regions. According to the Chinese Three Gradient Terrains^43^, Shannan Prefecture and Haidong Prefecture belong to the first terrain, Kelan, Dingxi, Datong, Jining, and Zhangjiakou belong to the second group, and Chifeng, Hinggan League, and Baicheng belong to the third group. Clearly, visible differences in black pigmentation were also found among the surface fungi of naked oat grains between the first terrain and the other terrains. This result thus indicated that SRD of naked oat characterized by black fungal discoloration mainly occurred in regions below 2000 m (elevation). Our previous reports showed that for grains of naked oat planted in Kelan, the infection level of *Alternaria* spp. on the black surface (38.7%) was five times as much as that on the normal surface (7.6%), while the infection level of *Davidiella* spp. on the black surface (3.9%) was similar to that on the normal surface (2.39%)^27^. *Alternaria* are dematiaceous fungi characterized by dark colonies ranging from gray to olive/brown^44^. Taking these results together, the black pathogen(s) causing SRD in eight of the regions examined may be *Alternaria* spp.

Further experimental results showed that the black pathogens cultured on PDA were *Alternaria* spp., which was consistent with the sequencing results; additionally, the greenhouse experiment results confirmed that *Alternaria* spp. was the cause of SRD of naked oat. Naked oat SRD differs from rice SRD^17,18^ in that it is associated with one pathogen. Oat leaf spot disease is only caused by *Alternaria alternata*^45–47^. *Alternaria* is one of the main mycotoxigenic fungal genera found in cereals worldwide^48^, causing diseases in over 400 host plants and postharvest spoilage of several crops^49,50^. The prevalence of this fungus in cereals indicates a high disease incidence, with more than 90% of the grains affected in the field^51^ and more than 50% of the grains affected in the greenhouse experiment in our study. Reports of cereal diseases characterized by leaf blackening and blight associated with *Alternaria* are continually published^16^, and cereal grains are constantly affected by *Alternaria* spp. and their toxins^51^. Although *Alternaria* toxins are often neglected in grains, possibly due to the lack of severe economic losses directly caused by the genus, recent studies have shown that their frequency and ability to produce a wide range of toxins is significant^10,16^. With governments attaching great importance to the sustainable development of the environment and agriculture and people’s concern for health, research and application of biological control are increasing. Microbial agents, botanical agents and resistance inducers are receiving increasing attention regarding their roles in the biocontrol of plant diseases caused by *Alternaria*^52^. Oat grains can be processed into many kinds of foods^53^. Thus, the finding that SRD of naked oat is caused by only *Alternaria* spp. presents an obvious advantage for the biological control of this disease.

## Conclusions

SRD has become an emerging naked oat disease in China, as losses caused by the disease have been regularly increasing over recent years. The pathogens of SRD have been investigated in many regions of Tibet, Shanxi Province, Gansu Province, Inner Mongolia, Qinghai Province and Hebei Province, China. Using metagenomic analysis, molecular techniques and field validation, one causal agent was identified as *Alternaria* spp., and this disease mainly occurred at altitudes below 2000 m. Our results can help improve the recognition, diagnosis, and management of this important disease. It is emphasized in this study that this disease is caused by a single pathogenic taxon, and further research into the application of antagonistic microbes against *Alternaria* spp. to prevent the occurrence of pre- and postharvest SRD of naked oat are required to ensure the production of certified oat materials.

## Materials and methods

### Plant material

Based on the Chinese oat and buckwheat industry, Bayou 13 plants have been grown for over five years in 10 different oat-producing regions (Datong City and Kelan County in Shanxi Province, Dingxi City in Gansu Province, Zhangjiakou City in Hebei Province, Baicheng City in Jilin Province, Shannan Prefecture in Tibet, Haidong Prefecture in Qinghai Province, and Chifeng City, Jining City, and Hinggan League in Inner Mongolia). The elevations, geographical coordinates, and meteorological data of the 10 regions are summarized in Supplementary Table S1. In Datong City, Dingxi City, Baicheng City, Chifeng City, and Hinggan League, Bayou 13 plants were sown in early April and harvested in early August. In Kelan County, Zhangjiakou City, Shannan Prefecture, Haidong Prefecture, and Jining City, Bayou 13 plants were sown in mid-late May and harvested in mid-September. In 2017, the seeds were sown in drills 3–5 cm deep, spaced ca. 25 cm apart and at an average rate of 450 seeds m^-2^ in these regions. Irrigation and fertilization were scheduled according to the oat requirements, soil storage capacity, and climate. The cropping system followed the guidelines of the National Oat and Buckwheat Industrial Technology System^6^. For each of the ten sites, we randomly selected three sampling plots; the plot dimensions were 10 m × 5.0 m, and each plot contained 20 rows. At the ripening stage of the Bayou 13 plants, 500 g of grains per plot for each region was collected by the Center for Agricultural Genetic Resources Research unit and stored in a polyethylene bag at − 80 °C.

### Collection of fungal pathogens on the grain surfaces and DNA extraction

The surfaces of oat grain samples were washed three times with phosphate-buffered saline (PBS), and the resultant pathogen-containing solutions were centrifuged at 15,000×*g* for 30 s. After the supernatant was discarded, total pathogen genomic DNA samples were extracted using Fast DNA SPIN extraction kits (MP Biomedicals, Santa Ana, CA, USA) following the manufacturer’s instructions and stored at − 20 °C prior to further analysis. The quantity and quality of extracted DNA were measured using a NanoDrop ND-1000 spectrophotometer (Thermo Fisher Scientific, Waltham, MA, USA) and agarose gel electrophoresis, respectively.

### 16S rDNA amplicon sequencing

PCR amplification of the fungal ITS1 region was performed using the forward primer ITS5 (5′-GGAAGTAAAAGTCGTAACAAGG-3′) and the reverse primer ITS2 (5′-GCTGCGTTCTTCATCGATGC-3′). Sample-specific 7-bp barcodes were incorporated into the primers for multiplex sequencing. PCR amplicons were purified with Agencourt AMPure Beads (Beckman Coulter, Indianapolis, IN, USA) and quantified using the PicoGreen dsDNA Assay Kit (Invitrogen, Carlsbad, CA, USA). After the individual quantification step, amplicons were pooled in equal amounts, and paired-end 2 × 300 bp sequencing was performed using the Illumina MiSeq platform with a MiSeq Reagent Kit v3 at Shanghai Personal Biotechnology Co., Ltd. (Shanghai, China).

### Sequence analysis

The Quantitative Insights Into Microbial Ecology (QIIME, v1.8.0) bioinformatic pipeline was employed to process the sequencing data, as previously described^54^. Briefly, raw sequencing reads with exact matches to the barcodes were assigned to respective samples and identified as valid sequences. The low-quality sequences were filtered with previously reported criteria^55,56^. Paired-end reads were assembled using FLASH^57^. After chimera detection, the remaining high-quality sequences were clustered into operational taxonomic units (OTUs) at 97% sequence identity by UCLUST^58^. A representative sequence was selected from each OTU using default parameters. OTUs were taxonomically classified by BLAST, and the representative sequence set was searched against the Unite Database^59^ using the best hits^60^. An OTU table was also generated to record the abundance and taxonomy of each OTU in each sample. OTUs containing less than 0.001% of total sequences across all samples were discarded. To minimize the difference in sequencing depth across samples, an averaged, rounded rarefied OTU table was generated by averaging 100 evenly resampled OTU subsets under 90% of the minimum sequencing depth for further analysis.

### Bioinformatics and statistical analysis

Sequence data analyses were mainly performed using QIIME and R packages (v3.2.0)^61^. OTU-level alpha diversity indices, such as the Chao1 richness estimator, abundance-based coverage estimator (ACE) metric, and Shannon diversity index, were calculated using the OTU table in QIIME. Taxon abundances at the genus level were statistically compared among samples by Metastats^62^ and visualized as violin plots. Cooccurrence analysis was performed by calculating Spearman’s rank correlations between predominant taxa. Correlations with |RHO|> 0.6 and *P* < 0.01 were visualized as a cooccurrence network using Cytoscape^63^.

### Culture of fungal pathogens from surfaces of oat grains from different ecological regions

From the samples of each region, 30 oat grains from the initial 500 g of grains were randomly selected. A conventional tissue separation method was used for isolating and culturing samples^64^. After the germ had been removed from each grain using a sterilized blade, the grains were disinfected in 75% ethanol for 30 s, washed three times with sterile water, placed on sterile filter paper to absorb the excess water on the grain surfaces, and evenly arranged using sterile forceps on potato dextrose agar (PDA) medium (potato [Kunming, China], 200 g l^−1^; glucose [Solarbio, Beijing, China], 20 g l^−1^; agar, 15 g l^−1^). For each ecological region, three PDA plates with 10 grains each were prepared, i.e., three parallel experiments were conducted. The loaded PDA plates were incubated in an upright position at 25 °C without light for 5 days^65^. The number of black grains was counted, and the morphology of the pathogens on the surfaces of naked oat grains was preliminarily observed using an Olympus BX53 microscope (Olympus, Japan).

### Identification of SRD-causing fungal pathogens on the surfaces of naked oat grains

According to the single spore isolation method^66^, single spores derived from all colonies around each grain from each region were selected from the culture medium. The purified strain was inoculated three to five times on new corn meal agar (CMA) medium (corn [Kunming, China], 20 g l^−1^; agar [Biofroxx, Einhausen, Germany], 15 g l^−1^). Then, the purified strain was inoculated on a PDA plate and stored at 4 °C^67^. After dark culture at 25 °C for 1 week, DNA was extracted from the fungal isolates, and sequencing was conducted by Shanghai Personal Biotechnology Co., Ltd. (Shanghai, China). By sequencing comparison using BLAST in the NCBI database, *Alternaria* spp. was identified as the main pathogen.

### Pathogenicity tests of *Alternaria* spp. on oat grains in the greenhouse

A pot cultivation trial using cultivar Bayou 13 plants collected from Kelan County was conducted in an environmentally controlled glass greenhouse at 25 °C and under a 12:12 LD photoperiod from early November 2018 to late May 2019. This experiment included three groups with three replicates each: the normal growth group (untreated), control group (sprayed with water containing 0.1% Tween-20), and experimental group (sprayed with an *Alternaria* spore suspension). Approximately 20 normal seeds were sown in a porcelain pot 40 cm in diameter and 30 cm in height. For each group, six pots were placed in a 3 m × 1.5 m plot as one repeat and arranged in two rows with interpot distances of 40 cm; in total, 18 pots and three hundred and sixty seeds were used for each group. Each group was at least five meters apart. The preserved *Alternaria* was cultured on PDA at 25 °C under a 12:12 LD photoperiod for 7 days. Subsequently, suspensions of conidia were prepared using sterile water containing 0.1% Tween-20, and the conidial suspensions were passed through two layers of a sterile cheesecloth to remove hyphal fragments^68^. Spore concentration was calculated using a hemocytometer and adjusted to 1 × 10^6^ spores/ml. During the maturation period, the experimental group was uniformly sprayed with the spore suspension, the control group was sprayed with sterile water containing 0.1% Tween-20, and the normal growth group was left untreated. Using a hand sprayer, 50 ml of spore suspension or 0.1% Tween-20 solution for each batch of six pots was directly sprayed on the rachises; each rachis was then put in a transparent plastic bag to prevent drying^69,70^. Spraying was performed once every morning, noon, and night for 2 weeks during the maturity stage. From each plot, 50 granules and 50 glumes were randomly selected, surface disinfected using 75% ethanol for 30 s, washed three times with sterile water, evenly arranged on CMA medium, and incubated at 25 °C. After 5 days, the number of black granules and glumes was recorded. The pathogens were isolated from the black areas of grains and glumes. The colony and conidial characteristics of the recovered pathogen were observed using an Olympus BX53 microscope (Olympus, Japan) and compared with those of the pathogen used for inoculation.

### Statistical analysis

The infestation ratio was calculated using the following equation:

$$ {\text{Infestation ratio (\%)}} = \frac{{\text{total number of infested grains or glumes}}}{{\text{total number of investigated grains or glumes}}} \times 100 $$

Data collected from the infestation ratio of grains, granules, or glumes were used for one-way analysis of variance (Fisher’s protected least significant difference), and normalized data were transformed using an arcsine square root function before analysis. Among ecological indices such as elevation, sunshine hours, temperature, rainfall, latitude and longitude from Supplementary Table S1, two models of key indices and two genera, *Alternaria* and *Davidiella* spp. were generated using stepwise backward selection^71^. All analyses were conducted in SPSS for Windows Version 16.0 (SPSS Inc., Chicago, Illinois, United States of America).

## Supplementary Information

Supplementary Information
