# Supplementary material for: Community structure of fungal pathogens causing spikelet rot disease of naked oat from different ecological regions of China
Source: Sci Rep. 2021 Jan 13;11:1243. doi: 10.1038/s41598-020-80273-6 (PMC7806892; doi:10.1038/s41598-020-80273-6)
Supplement: Supplementary file 1 — Supplementary Information [file 41598_2020_80273_MOESM1_ESM.docx]

Community structure of fungal pathogens causing spikelet rot disease of naked oat from different ecological regions of China

Longlong Liu *, Mingchuan Ma, Zhang Liu, Lijun Zhang & Jianping Zhou

Key Laboratory of Crop Gene Resources and Germplasm Enhancement on Loess Plateau, Center for Agricultural Genetic Resources Research, Shanxi Agricultural University, Taiyuan 030031, China

* Correspondence and requests for materials should be addressed to L. Liu. (email: lllong781211@sina.com)

**Running head**: Cause and distribution of naked oat spikelet rot disease

**Table S1**

| Ecological region | Elevation (m) | Geographical coordinates | Sunshine hours (h) | Temperature (°C) | Rainfall (mm) |
| --- | --- | --- | --- | --- | --- |
| Kelan County, Shanxi Province | 1404 | N38° 46′E111° 35′ | 2369.1 | 8.5–14.5 | 456 |
| Dingxi City, Gansu Province | 1920 | N35°32′E104° 42′ | 2100 | 9.5–15 | 494.2 |
| Datong City, Shanxi Province | 1067.0 | N40° 06′ E113° 20′ | 2821.6 | 7.7–14.7 | 384.0 |
| Chifeng City, Inner Mongolia | 605 | N41° 51′ E118°17′ | 2750 | 8.3–16 | 400 |
| Jining City, Inner Mongolia | 1400 | N41.3°59′ E114°07′ | 3000 | 5–12.1 | 360 |
| Hinggan League, Inner Mongolia | 286 | N46° 06′ E122° 03′ | 3901 | 11.5–18.5 | 416.7 |
| Zhangjiakou City, Hebei Province | 1393 | N41° 09′ E114° 42′ | 2392.3 | 10–17.5 | 234.8 |
| Baicheng City, Jilin Province | 153 | N45° 37′ E122° 49′ | 2885.8 | 6–14.9 | 399.8 |
| Shannan Prefecture, Tibet | 3660 | N29° 08′  E91° 47′ | 3005 | 10.5–15.5 | 394 |
| Haidong Prefecture, Qinghai Province | 2620 | N36° 28′ E102° 23′ | 2800 | 8–13.5 | 481 |

**Table S2**

| Species  Genera | Kelan County, Shanxi Province | Dingxi City, Gansu Province | Datong City, Shanxi Province | Chifeng City, Inner Mongolia | Jining City, Inner Mongolia | Hinggan League, Inner Mongolia | Zhangjiakou City, Hebei Province | Baicheng City, Jilin Province | Shannan Prefecture, Tibet | Haidong Prefecture, Qinghai Province |
| --- | --- | --- | --- | --- | --- | --- | --- | --- | --- | --- |
| Acremonium | *Acremonium alternatum* | *Acremonium alternatum* | *Acremonium alternatum* | *Acremonium alternatum* | *Acremonium alternatum* | *Acremonium alternatum* | *Acremonium alternatum* | *Acremonium alternatum* | *Acremonium alternatum* | *Acremonium alternatum* |
|  | *Acremonium brachypenium* | *Acremonium brachypenium* | *Acremonium brachypenium* | *Acremonium brachypenium* | *Acremonium brachypenium* | *Acremonium brachypenium* | *Acremonium brachypenium* | *Acremonium brachypenium* | *Acremonium brachypenium* | *Acremonium brachypenium* |
|  | *Acremonium fusidioides* | *Acremonium fusidioides* | *Acremonium fusidioides* | *Acremonium fusidioides* | *Acremonium fusidioides* | *Acremonium fusidioides* | *Acremonium fusidioides* | *Acremonium fusidioides* | *Acremonium fusidioides* | *Acremonium fusidioides* |
|  | *Acremonium implicatum* | *Acremonium implicatum* | *Acremonium implicatum* | *Acremonium implicatum* | *Acremonium implicatum* | *Acremonium implicatum* | *Acremonium implicatum* | *Acremonium implicatum* | *Acremonium implicatum* | *Acremonium implicatum* |
|  |  | *Acremonium nepalense* |  | *Acremonium nepalense* | *Acremonium nepalense* |  | *Acremonium nepalense* | *Acremonium nepalense* | *Acremonium nepalense* | *Acremonium nepalense* |
|  | *Acremonium sp* | *Acremonium sp* |  | *Acremonium sp* | *Acremonium sp* | *Acremonium sp* |  | *Acremonium sp* |  | *Acremonium sp* |
|  |  |  |  |  |  | *Acremonium sp OUCMBI101028* | *Acremonium sp OUCMBI101028* | *Acremonium sp OUCMBI101028* |  | *Acremonium sp OUCMBI101028* |
| Alternaria | *Alternaria eichhorniae* | *Alternaria eichhorniae* | *Alternaria eichhorniae* | *Alternaria eichhorniae* | *Alternaria eichhorniae* | *Alternaria eichhorniae* | *Alternaria eichhorniae* | *Alternaria eichhorniae* | *Alternaria eichhorniae* |  |
|  |  |  | *Alternaria leucanthemi* | *Alternaria leucanthemi* |  | *Alternaria leucanthemi* | *Alternaria leucanthemi* | *Alternaria leucanthemi* |  | *Alternaria leucanthemi* |
|  | *Alternaria sp* | *Alternaria sp* | *Alternaria sp* | *Alternaria sp* | *Alternaria sp* | *Alternaria sp* | *Alternaria sp* | *Alternaria sp* | *Alternaria sp* | *Alternaria sp* |
| Archaeorhizomyces |  | *Archaeorhizomyces sp* | *Archaeorhizomyces sp* | *Archaeorhizomyces sp* |  | *Archaeorhizomyces sp* |  |  | *Archaeorhizomyces sp* |  |
| Ascochyta | *Ascochyta sp* | *Ascochyta sp* | *Ascochyta sp* | *Ascochyta sp* | *Ascochyta sp* | *Ascochyta sp* | *Ascochyta sp* | *Ascochyta sp* | *Ascochyta sp* | *Ascochyta sp* |
| Aspergillus | *Aspergillus cibarius* | *Aspergillus cibarius* | *Aspergillus cibarius* | *Aspergillus cibarius* | *Aspergillus cibarius* | *Aspergillus cibarius* | *Aspergillus cibarius* | *Aspergillus cibarius* | *Aspergillus cibarius* | *Aspergillus cibarius* |
|  |  | *Aspergillus ochraceus* | *Aspergillus ochraceus* | *Aspergillus ochraceus* |  | *Aspergillus ochraceus* |  | *Aspergillus ochraceus* | *Aspergillus ochraceus* | *Aspergillus ochraceus* |
|  |  |  | *Aspergillus subversicolor* | *Aspergillus subversicolor* | *Aspergillus subversicolor* | *Aspergillus subversicolor* | *Aspergillus subversicolor* | *Aspergillus subversicolor* | *Aspergillus subversicolor* | *Aspergillus subversicolor* |
| Aureobasidium | *Aureobasidium pullulans* | *Aureobasidium pullulans* | *Aureobasidium pullulans* | *Aureobasidium pullulans* | *Aureobasidium pullulans* | *Aureobasidium pullulans* | *Aureobasidium pullulans* | *Aureobasidium pullulans* | *Aureobasidium pullulans* | *Aureobasidium pullulans* |
| Bipolaris | *Bipolaris maydis* | *Bipolaris maydis* | *Bipolaris maydis* | *Bipolaris maydis* | *Bipolaris maydis* | *Bipolaris maydis* | *Bipolaris maydis* | *Bipolaris maydis* | *Bipolaris maydis* | *Bipolaris maydis* |
|  | *Bipolaris microstegii* | *Bipolaris microstegii* |  | *Bipolaris microstegii* | *Bipolaris microstegii* | *Bipolaris microstegii* |  | *Bipolaris microstegii* |  |  |
| Botrytis | *Botrytis caroliniana* | *Botrytis caroliniana* | *Botrytis caroliniana* | *Botrytis caroliniana* | *Botrytis caroliniana* | *Botrytis caroliniana* | *Botrytis caroliniana* | *Botrytis caroliniana* | *Botrytis caroliniana* | *Botrytis caroliniana* |
| Bullera | *Bullera sp VY_86* | *Bullera sp VY_86* | *Bullera sp VY_86* | *Bullera sp VY_86* | *Bullera sp VY_86* | *Bullera sp VY_86* | *Bullera sp VY_86* | *Bullera sp VY_86* | *Bullera sp VY_86* | *Bullera sp VY_86* |
|  | *Bullera unica* | *Bullera unica* | *Bullera unica* | *Bullera unica* | *Bullera unica* | *Bullera unica* |  | *Bullera unica* |  | *Bullera unica* |
| Chalastospora |  | *Chalastospora ellipsoidea* | *Chalastospora ellipsoidea* | *Chalastospora ellipsoidea* | *Chalastospora ellipsoidea* | *Chalastospora ellipsoidea* | *Chalastospora ellipsoidea* | *Chalastospora ellipsoidea* | *Chalastospora ellipsoidea* | *Chalastospora ellipsoidea* |
| Cladosporium | *Cladosporium arthropodii* |  | *Cladosporium arthropodii* | *Cladosporium arthropodii* | *Cladosporium arthropodii* |  |  | *Cladosporium arthropodii* |  |  |
|  | *Cladosporium grevilleae* | *Cladosporium grevilleae* | *Cladosporium grevilleae* | *Cladosporium grevilleae* | *Cladosporium grevilleae* | *Cladosporium grevilleae* | *Cladosporium grevilleae* | *Cladosporium grevilleae* | *Cladosporium grevilleae* | *Cladosporium grevilleae* |
| Cryptococcus | *Cryptococcus albidus* | *Cryptococcus albidus* | *Cryptococcus albidus* | *Cryptococcus albidus* | *Cryptococcus albidus* |  | *Cryptococcus albidus* | *Cryptococcus albidus* | *Cryptococcus albidus* | *Cryptococcus albidus* |
|  | *Cryptococcus aureus* |  | *Cryptococcus aureus* | *Cryptococcus aureus* |  | *Cryptococcus aureus* |  | *Cryptococcus aureus* | *Cryptococcus aureus* |  |
|  | *Cryptococcus sp SM13L02* | *Cryptococcus sp SM13L02* |  | *Cryptococcus sp SM13L02* | *Cryptococcus sp SM13L02* | *Cryptococcus sp SM13L02* | *Cryptococcus sp SM13L02* | *Cryptococcus sp SM13L02* | *Cryptococcus sp SM13L02* | *Cryptococcus sp SM13L02* |
|  | *Cryptococcus victoriae* | *Cryptococcus victoriae* | *Cryptococcus victoriae* | *Cryptococcus victoriae* | *Cryptococcus victoriae* | *Cryptococcus victoriae* | *Cryptococcus victoriae* | *Cryptococcus victoriae* | *Cryptococcus victoriae* | *Cryptococcus victoriae* |
|  | *Cryptococcus wieringae* | *Cryptococcus wieringae* | *Cryptococcus wieringae* | *Cryptococcus wieringae* | *Cryptococcus wieringae* | *Cryptococcus wieringae* | *Cryptococcus wieringae* | *Cryptococcus wieringae* | *Cryptococcus wieringae* | *Cryptococcus wieringae* |
| Davidiella | *Davidiella tassiana* | *Davidiella tassiana* | *Davidiella tassiana* | *Davidiella tassiana* | *Davidiella tassiana* | *Davidiella tassiana* | *Davidiella tassiana* | *Davidiella tassiana* | *Davidiella tassiana* | *Davidiella tassiana* |
| Dermatocarpon | *Dermatocarpon luridum var luridum* | *Dermatocarpon luridum var luridum* |  | *Dermatocarpon luridum var luridum* |  | *Dermatocarpon luridum var luridum* |  | *Dermatocarpon luridum var luridum* |  | *Dermatocarpon luridum var luridum* |
| Didymella | *Didymella exigua* | *Didymella exigua* | *Didymella exigua* | *Didymella exigua* | *Didymella exigua* | *Didymella exigua* | *Didymella exigua* | *Didymella exigua* | *Didymella exigua* | *Didymella exigua* |
|  | *Didymella sp* | *Didymella sp* | *Didymella sp* | *Didymella sp* | *Didymella sp* |  | *Didymella sp* | *Didymella sp* | *Didymella sp* | *Didymella sp* |
| Dioszegia |  | *Dioszegia fristingensis* | *Dioszegia fristingensis* | *Dioszegia fristingensis* |  |  |  | *Dioszegia fristingensis* | *Dioszegia fristingensis* | *Dioszegia fristingensis* |
|  | *Dioszegia hungarica* | *Dioszegia hungarica* |  | *Dioszegia hungarica* | *Dioszegia hungarica* | *Dioszegia hungarica* | *Dioszegia hungarica* |  | *Dioszegia hungarica* | *Dioszegia hungarica* |
| Dothidea |  |  |  |  |  | *Dothidea hippophaeos* |  |  |  | *Dothidea hippophaeos* |
| Fusarium |  | *Fusarium sp E10021c* |  | *Fusarium sp E10021c* | *Fusarium sp E10021c* | *Fusarium sp E10021c* |  | *Fusarium sp E10021c* |  |  |
|  | *Fusarium tricinctum* | *Fusarium tricinctum* | *Fusarium tricinctum* | *Fusarium tricinctum* | *Fusarium tricinctum* | *Fusarium tricinctum* | *Fusarium tricinctum* | *Fusarium tricinctum* | *Fusarium tricinctum* | *Fusarium tricinctum* |
| Gibberella | *Gibberella zeae* | *Gibberella zeae* | *Gibberella zeae* | *Gibberella zeae* | *Gibberella zeae* | *Gibberella zeae* | *Gibberella zeae* | *Gibberella zeae* | *Gibberella zeae* | *Gibberella zeae* |
| Gloiothele |  |  |  | *Gloiothele citrina* |  |  |  |  |  |  |
| Hannaella |  |  | *Hannaella luteola* | *Hannaella luteola* | *Hannaella luteola* |  | *Hannaella luteola* | *Hannaella luteola* |  |  |
|  | *Hannaella siamensis* |  | *Hannaella siamensis* | *Hannaella siamensis* | *Hannaella siamensis* | *Hannaella siamensis* | *Hannaella siamensis* | *Hannaella siamensis* | *Hannaella siamensis* |  |
| Lecythophora |  |  |  | *Lecythophora sp* |  | *Lecythophora sp* |  | *Lecythophora sp* | *Lecythophora sp* |  |
| Malbranchea |  |  |  | *Malbranchea sp* |  |  |  |  |  |  |
| Microdochium | *Microdochium bolleyi* | *Microdochium bolleyi* | *Microdochium bolleyi* | *Microdochium bolleyi* | *Microdochium bolleyi* | *Microdochium bolleyi* | *Microdochium bolleyi* | *Microdochium bolleyi* | *Microdochium bolleyi* |  |
| Monographella |  |  |  | *Monographella nivalis* |  |  |  |  | *Monographella nivalis* | *Monographella nivalis* |
| Mortierella |  |  |  | *Mortierella camargensis* |  |  |  |  | *Mortierella camargensis* |  |
| Peltaster |  |  |  | *Peltaster cerophilus* | *Peltaster cerophilus* | *Peltaster cerophilus* |  | *Peltaster cerophilus* | *Peltaster cerophilus* |  |
| Penicillium |  |  | *Penicillium bialowiezense* | *Penicillium bialowiezense* | *Penicillium bialowiezense* |  | *Penicillium bialowiezense* | *Penicillium bialowiezense* |  |  |
|  | *Penicillium charlesii* |  |  |  |  |  |  | *Penicillium charlesii* |  |  |
|  |  |  | *Penicillium polonicum* | *Penicillium polonicum* | *Penicillium polonicum* | *Penicillium polonicum* |  | *Penicillium polonicum* | *Penicillium polonicum* |  |
|  | *Penicillium sp K6_5* |  | *Penicillium sp K6_5* | *Penicillium sp K6_5* | *Penicillium sp K6_5* |  |  | *Penicillium sp K6_5* |  |  |
| Phaeoacremonium |  | *Phaeoacremonium hungaricum* |  | *Phaeoacremonium hungaricum* | *Phaeoacremonium hungaricum* | *Phaeoacremonium hungaricum* |  | *Phaeoacremonium hungaricum* | *Phaeoacremonium hungaricum* |  |
| Phaeosphaeria | *Phaeosphaeria sp* | *Phaeosphaeria sp* |  | *Phaeosphaeria sp* | *Phaeosphaeria sp* |  | *Phaeosphaeria sp* |  |  |  |
| Phoma | *Phoma calidophila* | *Phoma calidophila* | *Phoma calidophila* | *Phoma calidophila* | *Phoma calidophila* | *Phoma calidophila* | *Phoma calidophila* | *Phoma calidophila* | *Phoma calidophila* | *Phoma calidophila* |
| Plenodomus |  | *Plenodomus chrysanthemi* |  | *Plenodomus chrysanthemi* | *Plenodomus chrysanthemi* | *Plenodomus chrysanthemi* | *Plenodomus chrysanthemi* |  |  |  |
| Pleospora | *Pleospora herbarum* | *Pleospora herbarum* | *Pleospora herbarum* | *Pleospora herbarum* | *Pleospora herbarum* | *Pleospora herbarum* | *Pleospora herbarum* | *Pleospora herbarum* | *Pleospora herbarum* | *Pleospora herbarum* |
| Pyrenophora | *Pyrenophora tritici_repentis* |  | *Pyrenophora tritici_repentis* | *Pyrenophora tritici_repentis* |  |  | *Pyrenophora tritici_repentis* | *Pyrenophora tritici_repentis* |  |  |
| Radulidium | *Radulidium subulatum* | *Radulidium subulatum* | *Radulidium subulatum* | *Radulidium subulatum* | *Radulidium subulatum* | *Radulidium subulatum* | *Radulidium subulatum* | *Radulidium subulatum* |  | *Radulidium subulatum* |
| Sarocladium |  |  |  |  |  | *Sarocladium glaucum* |  | *Sarocladium glaucum* |  |  |
|  |  |  |  |  |  |  |  | *Sarocladium sp* |  |  |
|  | *Sarocladium strictum* | *Sarocladium strictum* | *Sarocladium strictum* | *Sarocladium strictum* | *Sarocladium strictum* | *Sarocladium strictum* | *Sarocladium strictum* | *Sarocladium strictum* | *Sarocladium strictum* | *Sarocladium strictum* |
| Sepedonium |  |  |  |  |  | *Sepedonium laevigatum* |  | *Sepedonium laevigatum* |  |  |
| Setosphaeria |  |  | *Setosphaeria rostrata* | *Setosphaeria rostrata* | *Setosphaeria rostrata* | *Setosphaeria rostrata* | *Setosphaeria rostrata* | *Setosphaeria rostrata* | *Setosphaeria rostrata* | *Setosphaeria rostrata* |
| Sphaerulina |  | *Sphaerulina tirolensis* |  | *Sphaerulina tirolensis* | *Sphaerulina tirolensis* | *Sphaerulina tirolensis* | *Sphaerulina tirolensis* | *Sphaerulina tirolensis* |  |  |
| Sporobolomyces | *Sporobolomyces oryzicola* | *Sporobolomyces oryzicola* |  | *Sporobolomyces oryzicola* | *Sporobolomyces oryzicola* | *Sporobolomyces oryzicola* |  | *Sporobolomyces oryzicola* |  |  |
|  |  |  |  |  | *Sporobolomyces ruberrimus* | *Sporobolomyces ruberrimus* |  | *Sporobolomyces ruberrimus* |  |  |
| Tiarosporella | *Tiarosporella urbis-rosarum* | *Tiarosporella urbis-rosarum* | *Tiarosporella urbis-rosarum* | *Tiarosporella urbis-rosarum* | *Tiarosporella urbis-rosarum* | *Tiarosporella urbis-rosarum* | *Tiarosporella urbis-rosarum* | *Tiarosporella urbis-rosarum* | *Tiarosporella urbis-rosarum* | *Tiarosporella urbis-rosarum* |
| Udeniomyces |  | *Udeniomyces pannonicus* |  | *Udeniomyces pannonicus* | *Udeniomyces pannonicus* |  |  |  | *Udeniomyces pannonicus* | *Udeniomyces pannonicus* |
| unidentified |  |  |  | *Archaeorhizomycetales sp* |  |  |  |  |  |  |
|  | *Ascomycota sp* | *Ascomycota sp* | *Ascomycota sp* | *Ascomycota sp* | *Ascomycota sp* | *Ascomycota sp* | *Ascomycota sp* | *Ascomycota sp* | *Ascomycota sp* | *Ascomycota sp* |
|  | *Basidiomycota sp* | *Basidiomycota sp* |  | *Basidiomycota sp* |  | *Basidiomycota sp* |  | *Basidiomycota sp* |  |  |
|  | *Dothideomycetes sp* | *Dothideomycetes sp* | *Dothideomycetes sp* | *Dothideomycetes sp* | *Dothideomycetes sp* | *Dothideomycetes sp* | *Dothideomycetes sp* | *Dothideomycetes sp* | *Dothideomycetes sp* |  |
|  |  |  |  |  |  |  |  | *Eurotiales sp* |  |  |
|  |  | *Exobasidiomycetes sp* |  | *Exobasidiomycetes sp* | *Exobasidiomycetes sp* | *Exobasidiomycetes sp* | *Exobasidiomycetes sp* | *Exobasidiomycetes sp* |  |  |
|  | *Filobasidiales sp* | *Filobasidiales sp* | *Filobasidiales sp* | *Filobasidiales sp* | *Filobasidiales sp* | *Filobasidiales sp* | *Filobasidiales sp* | *Filobasidiales sp* | *Filobasidiales sp* | *Filobasidiales sp* |
|  | *Fungi sp* | *Fungi sp* | *Fungi sp* | *Fungi sp* | *Fungi sp* | *Fungi sp* | *Fungi sp* | *Fungi sp* | *Fungi sp* | *Fungi sp* |
|  |  |  |  | *Geoglossales sp* |  |  |  |  |  |  |
|  |  |  |  | *Helotiales sp* |  |  |  | *Helotiales sp* | *Helotiales sp* | *Helotiales sp* |
|  | *Hypocreales sp* | *Hypocreales sp* | *Hypocreales sp* | *Hypocreales sp* | *Hypocreales sp* | *Hypocreales sp* | *Hypocreales sp* | *Hypocreales sp* | *Hypocreales sp* | *Hypocreales sp* |
|  | *Leotiomycetes sp* | *Leotiomycetes sp* |  |  |  |  |  |  |  | *Leotiomycetes sp* |
|  |  |  | *Mycosphaerellaceae sp* |  | *Mycosphaerellaceae sp* | *Mycosphaerellaceae sp* |  |  | *Mycosphaerellaceae sp* | *Mycosphaerellaceae sp* |
|  | *Nectriaceae sp* | *Nectriaceae sp* | *Nectriaceae sp* | *Nectriaceae sp* | *Nectriaceae sp* | *Nectriaceae sp* | *Nectriaceae sp* | *Nectriaceae sp* | *Nectriaceae sp* | *Nectriaceae sp* |
|  | *Phaeosphaeriaceae sp* | *Phaeosphaeriaceae sp* | *Phaeosphaeriaceae sp* | *Phaeosphaeriaceae sp* |  | *Phaeosphaeriaceae sp* | *Phaeosphaeriaceae sp* | *Phaeosphaeriaceae sp* | *Phaeosphaeriaceae sp* | *Phaeosphaeriaceae sp* |
|  | *Plantae sp* | *Plantae sp* | *Plantae sp* | *Plantae sp* |  | *Plantae sp* | *Plantae sp* |  | *Plantae sp* | *Plantae sp* |
|  | *Pleosporaceae sp* | *Pleosporaceae sp* | *Pleosporaceae sp* | *Pleosporaceae sp* | *Pleosporaceae sp* | *Pleosporaceae sp* | *Pleosporaceae sp* | *Pleosporaceae sp* | *Pleosporaceae sp* | *Pleosporaceae sp* |
|  | *Pleosporales sp* | *Pleosporales sp* | *Pleosporales sp* | *Pleosporales sp* | *Pleosporales sp* | *Pleosporales sp* | *Pleosporales sp* | *Pleosporales sp* | *Pleosporales sp* | *Pleosporales sp* |
|  |  | *Poaceae sp* | *Poaceae sp* | *Poaceae sp* |  | *Poaceae sp* |  |  | *Poaceae sp* |  |
|  | *Sordariomycetes sp* | *Sordariomycetes sp* | *Sordariomycetes sp* | *Sordariomycetes sp* | *Sordariomycetes sp* | *Sordariomycetes sp* | *Sordariomycetes sp* | *Sordariomycetes sp* | *Sordariomycetes sp* | *Sordariomycetes sp* |
|  | *Sporidiobolales sp* | *Sporidiobolales sp* | *Sporidiobolales sp* | *Sporidiobolales sp* | *Sporidiobolales sp* | *Sporidiobolales sp* | *Sporidiobolales sp* | *Sporidiobolales sp* | *Sporidiobolales sp* | *Sporidiobolales sp* |
|  | *Tremellomycetes sp* | *Tremellomycetes sp* | *Tremellomycetes sp* | *Tremellomycetes sp* | *Tremellomycetes sp* | *Tremellomycetes sp* | *Tremellomycetes sp* | *Tremellomycetes sp* | *Tremellomycetes sp* | *Tremellomycetes sp* |
|  |  | *Tubeufiaceae sp* |  | *Tubeufiaceae sp* |  |  |  |  |  |  |
|  |  | *Ustilaginaceae sp* |  | *Ustilaginaceae sp* |  | *Ustilaginaceae sp* | *Ustilaginaceae sp* |  | *Ustilaginaceae sp* |  |
| Verticillium |  |  |  | *Verticillium albo-atrum* | *Verticillium albo-atrum* |  | *Verticillium albo-atrum* |  |  | *Verticillium albo-atrum* |
| Wallemia | *Wallemia sebi* | *Wallemia sebi* | *Wallemia sebi* | *Wallemia sebi* | *Wallemia sebi* | *Wallemia sebi* | *Wallemia sebi* | *Wallemia sebi* | *Wallemia sebi* | *Wallemia sebi* |
| No blast hit |  |  |  |  |  |  |  |  |  |  |


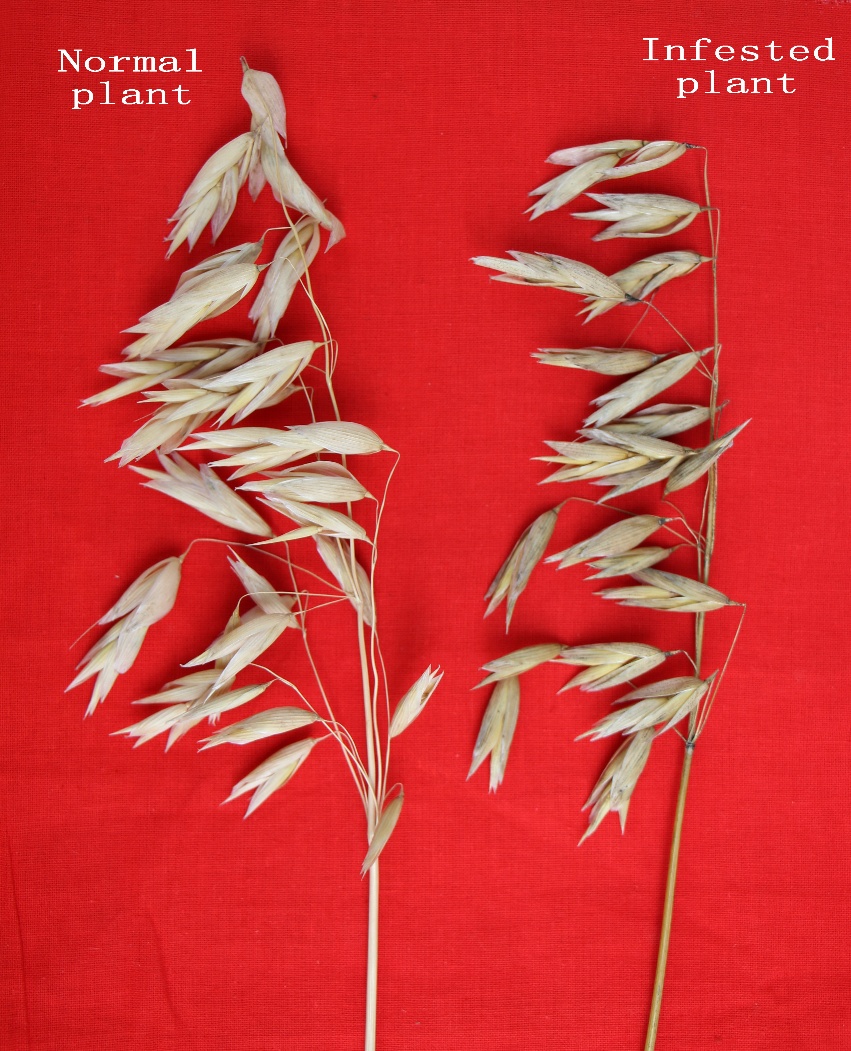


**Figure S1**


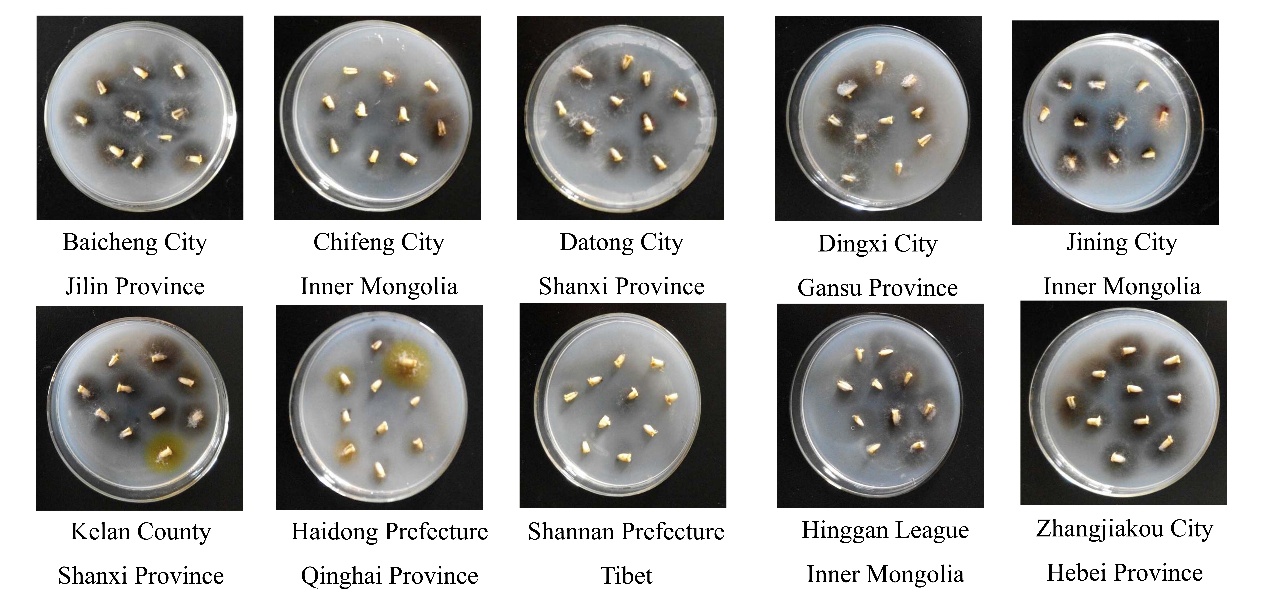


**Figure S2**
